# Audiometric changes following oral versus local gel foam steroid administration after stapedectomy in otosclerosis patients: a retrospective comparative study

**DOI:** 10.3389/fmed.2026.1782153

**Published:** 2026-02-19

**Authors:** Alaa Safia, Raed Farhat, Uday Abd Elhadi, Samraa Zahwa, Doris Jubran, Saleh Amara, Shlomo Merchavy, Saqr Massoud

**Affiliations:** 1 Department of Otolaryngology, Otology Unit, Ziv Medical Center, Safed, Israel; 2 Department of Speech, Language, and Audiology, Ziv Medical Center, Safed, Israel

**Keywords:** air–bone gap, bone-conduction thresholds, otosclerosis, postoperative complications, stapedectomy, steroid therapy

## Abstract

This study compared the effects of two postoperative steroid administration routes—oral prednisone versus local intraoperative dexamethasone gel foam—on hearing outcomes and complications following primary stapedectomy for otosclerosis. In this retrospective comparative study, 76 consecutive patients who underwent primary stapedectomy by a single senior surgeon at a tertiary center between 2016 and 2024 were included. Patients received either oral prednisone (50 mg daily for 5 days postoperatively; *n* = 37) or local dexamethasone (8 mg applied intraoperatively on gel foam; *n* = 39). Pre- and postoperative audiometric data, demographic characteristics, and postoperative complications were analyzed. Audiometry was performed 6–12 weeks after surgery, assessing air-conduction (AC), bone-conduction (BC), and air-bone gap (ABG) thresholds. Both groups demonstrated significant postoperative AC improvement and comparable ABG closure, with no significant differences in overall AC gain or ABG reduction. BC thresh-olds improved in both groups; however, greater BC gain was observed in the oral steroid group. Postoperative tinnitus and infections occurred more frequently in the local steroid group, while vertigo rates were similar between groups. In conclusion, oral and local steroid administration following primary stapedectomy were associated with comparable improvements in air-conduction and air–bone gap outcomes. Although greater bone-conduction recovery and fewer postoperative complications were observed in the oral steroid group, these findings should be interpreted cautiously given the retrospective design and limited sample size.

## 1 Introduction

Otosclerosis is a pathologic condition of the otic capsule characterized by abnormal bone remodeling that leads to stapes footplate fixation, impaired ossicular mobility and consequent conductive or mixed hearing loss (1). Surgical intervention via stapedectomy or stapedotomy remains the gold standard for restoring sound transmission in patients with stapes fixation (1, 2). Although modern techniques demonstrate excellent audiologic outcomes—with many series reporting air–bone gap (ABG) closure to ≤10 dB in over half of primary surgeries—the procedure remains not without risk, particularly regarding sensorineural hearing change, vestibular symptoms and tinnitus (3, 4).

In an effort to optimize post-operative recovery and minimize inner-ear trauma, attention has turned toward adjunctive therapies, especially corticosteroids. Glucocorticoids exert anti-inflammatory, anti-fibrotic and immunomodulatory effects, and their use in various inner-ear disorders—including sudden sensorineural hearing loss and Ménière’s disease—has been well documented (5, 6). Although the role of steroids in the context of stapes surgery is less clearly defined, several retrospective analyses suggest a potential benefit of systemic peri-operative corticosteroids in improving hearing thresholds and reducing complications. For example, a retrospective cohort of 84 CO_2_ laser-assisted stapedotomies reported superior postoperative AC thresholds in patients who received peri-operative steroids, though the difference did not reach statistical significance (7). More recently, a series evaluating local dexamethasone applied via the middle ear reported better ABG closure and BC improvements at 1,000–4,000 Hz in the steroid-treated arm (8).

Despite these promising signals, the optimal route, timing, dose and duration of steroid therapy in stapedectomy remain unestablished. Comparisons between systemic versus local (gel-foam or intratympanic) delivery are scarce, and existing studies are limited by heterogeneous designs and small sample sizes. Notwithstanding the consistency of good conductive outcomes following stapes surgery, the incremental impact of adjunctive steroid regimen on bone-conduction preservation, ABG closure, and complication rates is worth further investigation. Accordingly, the purpose of the present study is to retrospectively compare oral versus local gel-foam steroid administration in patients undergoing primary stapedectomy for otosclerosis, with specific focus on hearing thresholds (air-conduction (AC), bone-conduction (BC), ABG) at multiple frequencies and early postoperative complications.

## 2 Materials and methods

### 2.1 Study design and setting

This single-center retrospective comparative study included patients who underwent primary stapedectomy for otosclerosis between January 2016 and December 2024. The primary objective was to evaluate the effect of post-operative steroid administration route—oral versus local gel foam injection—on hearing outcomes and post-operative complications. Institutional Review Board approval was waived for this study due to its retrospective design, which involved analysis of existing medical records without direct patient contact or intervention. All procedures were conducted in accordance with the ethical standards of our center and the principles of the Declaration of Helsinki.

### 2.2 Study population

The study population comprised 76 consecutive patients diagnosed with otosclerosis who underwent primary stapedectomy during the study period. All procedures were performed by a single senior otologic surgeon to ensure consistency in surgical technique and minimize inter-operator variability. Patients were eligible for inclusion if they had complete pre-operative and post-operative audiometric outcome data at the specified time points and no history of revision stapedectomy. Cases with missing audiometric outcome data were excluded. Patients were also excluded if there was evidence of concomitant middle ear pathology or systemic illness potentially affecting auditory recovery. Minor missing demographic or clinical variables did not preclude inclusion, and analyses were performed using available-case data for those variables.

### 2.3 Surgical procedure

All operations were carried out using a standardized stapedectomy technique. Following elevation of the tympanomeatal flap, the sclerotic stapes footplate was carefully removed from the oval window, and a prosthesis was inserted to re-establish ossicular continuity. At the conclusion of the procedure, patients were assigned to one of two groups based on the route of steroid administration. In the local steroid group, a single ampoule of Dexacort® (8 mg dexamethasone) was injected onto a small piece of gel foam, which was placed around the oval window niche. In the oral steroid group, patients were prescribed prednisone 50 mg orally once daily for five consecutive days after surgery. All other perioperative management procedures, including anesthesia, antibiotic prophylaxis, and post-operative care, were standardized across both groups. Treatment allocation was not randomized and may therefore be subject to selection bias.

### 2.4 Data collection

Patient data were extracted from electronic medical records, including demographic variables (age, sex), prior hearing aid use, and baseline hearing status. Hearing status was classified according to the World Health Organization (WHO) hearing impairment grading system, where mild impairment corresponded to thresholds between 26 and 40 dB, moderate impairment to 41–55 dB, and moderately severe impairment to 56–70 dB (9). Audiometric parameters included AC, BC, and ABG thresholds at 250, 500, 1,000, 2,000, and 4,000 Hz. The pure-tone average (PTA) was calculated as the mean threshold at 0.5, 1, 2, and 4 kHz. Post-operative audiometric evaluation was routinely performed within 6–12 weeks after surgery. Secondary variables included post-operative complications such as infection, tinnitus, and dizziness.

### 2.5 Outcome measures

The primary outcome of the study was the change in air-conduction thresholds and air–bone gap closure following stapedectomy. Secondary outcomes included changes in bone-conduction thresholds and the incidence of post-operative complications in each steroid administration group.

### 2.6 Statistical analysis

All analyses were conducted using STATA version 18.0 (Stata Corp, College Station, TX, USA). Continuous variables were expressed as mean ± standard deviation (SD), while categorical variables were summarized as frequencies and percentages. Between-group comparisons of pre-operative, post-operative, and change (Δ) values were performed using independent-samples *t*-tests, and within-group (pre- versus post-operative) comparisons were evaluated using paired *t*-tests. Categorical variables were analyzed using the *χ*^2^ test or Fisher’s exact test when appropriate. A two-tailed *p*-value < 0.05 was considered statistically significant.

## 3 Results

### 3.1 Baseline characteristics

A total of 76 patients were included in the analysis, comprising 37 in the oral steroid group and 39 in the local gel foam steroid group (Table 1). The mean age was significantly higher in the oral group than in the local group (50.8 ± 11.3 vs. 40.6 ± 13.9 years, *p* = 0.0008). The mean body-mass index did not differ significantly between groups (27.0 ± 3.8 vs. 25.9 ± 4.1 kg/m^2^, *p* = 0.25). Duration of hearing loss was notably longer in the oral group (9.8 ± 5.4 years) compared with the local group (5.4 ± 2.7 years, *p* < 0.0001).

**Table 1:** Baseline demographic and clinical characteristics of patients by steroid administration group.

Characteristic	Per oral	Local	*p*-value
	*N* = 37	*N* = 39	
Age—yr	*n* = 37	*N* = 39	0.0008
Mean (SD)	50.78 (11.32)	40.56 (13.90)	
BMI	*n* = 36	*N* = 39	0.2475
Mean (SD)	27 (3.78)	25.94 (4.12)	
Hearing loss—yr	*n* = 37	*N* = 39	<0.0001
Mean (SD)	9.84 (5.35)	5.41 (2.65)	
Gender—no. (%)	*n* = 37	*N* = 39	0.438
Male	12 (32.43%)	16 (41.03%)	
Female	25 (67.57%)	23 (58.97%)	
Diabetes	*n* = 36	*n* = 39	0.415
Distribution—no. (%)	6 (16.67%)	4 (10.26%)	
Hypertension	*n* = 36	*n* = 39	0.82
Distribution—no. (%)	4 (11.11%)	5 (12.82%)	
Smoking	*n* = 36	*N* = 39	0.036
Distribution—no. (%)	6 (16.67%)	1 (2.56%)	
Alcohol drinking	*n* = 36	*N* = 39	-
Distribution—no. (%)	0 (0%)	0 (0%)	
Prior hearing intervention	*n* = 37	*N* = 39	0.509
Distribution—no. (%)	17 (45.95%)	15 (38.46%)	
Operated side—no. (%)	*n* = 37	*N* = 39	0.373
Right	20 (54.05%)	25 (64.10%)	
Left	17 (45.95%)	14 (35.90%)	
Baseline hearing status—no. (%)	*n* = 37	*N* = 39	0.035
Mild	9 (24.32%)	2 (5.13%)	
Moderate	17 (45.95%)	27 (69.23%)	
Severe	11 (29.73%)	10 (25.64%)	
Postoperative outcomes—no. (%)	*n* = 36	*N* = 39	
Tinnitus	3 (8.33%)	17 (43.59%)	0.001
Vertigo	9 (25%)	10 (25.64%)	0.949
Infection	0 (0%)	4 (10.26%)	0.048

no., number of cases; SD, standard deviation; *n*, number of cases with available data; BMI, body mass index; yr, year.

Sex distribution was comparable, with females representing 67.6% of the oral group and 59.0% of the local group (*p* = 0.44). The prevalence of diabetes and hypertension was similar across groups (*p* = 0.42 and *p* = 0.82, respectively). Smoking history was more frequent in the oral group (16.7%) than in the local group (2.6%, *p* = 0.036). None of the patients reported alcohol consumption. Prior hearing-aid use did not differ significantly between groups (46.0% vs. 38.5%, *p* = 0.51), nor did the operated side (*p* = 0.37).

Baseline audiometric severity was slightly worse in the local steroid group: moderate hearing loss predominated (69.2% vs. 46.0%), whereas mild hearing loss was more frequent in the oral group (24.3% vs. 5.1%, *p* = 0.035).

Post-operative complications differed significantly between groups. The incidence of tinnitus was markedly higher in the local group than in the oral group (43.6% vs. 8.3%, *p* = 0.001). Post-operative vertigo occurred in roughly one-quarter of both groups (*p* = 0.95). Infection was observed only in the local group (10.3%) and in none of the oral steroid cases (*p* = 0.048).

### 3.2 Air-conduction thresholds

Pre-operative AC thresholds were similar between groups at all tested frequencies (Table 2). Following surgery, substantial improvement was observed in both groups across all frequencies, with no statistically significant differences in the post-operative values or in the mean change (*Δ* = post-operative – pre-operative).

**Table 2:** Comparison of pre-operative, post-operative, and Δ (improvement) air-conduction thresholds between oral and local steroid groups.

Characteristic	Preoperative			Postoperative			Delta (Postop − Preop)		
	Per oral	Local	*p*	Per oral	Local	*p*	Per oral	Local	*p*
AC (250 Hz)	57.7 (12.96)	57.85 (13.66)	0.9627	26.70 (11.07)	25.21 (9.01)	0.5188	−31 (16.59)	−32.64 (15.19)	0.6539
AC (500 Hz)	61.59 (14.30)	58.36 (15.09)	0.3410	22.95 (12.40)	22.82 (10.22)	0.9617	−38.65 (18.04)	−35.54 (15.37)	0.4203
AC (1,000 Hz)	60.05 (16.96)	58.33 (14.54)	0.6357	24.92 (10.30)	23.64 (12.06)	0.6218	−35.14 (18.47)	−34.69 (14.46)	0.9074
AC (2,000 Hz)	53.92 (18.68)	50.10 (16.56)	0.3485	25.70 (11.91)	23.08 (12.71)	0.3563	−28.22 (23.26)	−27.03 (17.39)	0.8005
AC (4,000 Hz)	56.92 (17.95)	54.26 (21.55)	0.5613	27.57 (13.86)	27.31 (17.69)	0.9436	−29.35 (21.27)	−26.95 (27.21)	0.6704
AC (PTA)	60.43 (14.68)	56.20 (14.07)	0.2030	23.21 (11.83)	20.47 (11.64)	0.3127	−37.23 (18.14)	−35.73 (15.79)	0.7015

Data are presented as means (standard deviation) using the *t*-test for between group comparison. AC, air conduction; PTA, pure-tone average; *p*, *p*-value.

At 500 Hz, mean AC improved from 61.6 ± 14.3 dB to 23.0 ± 12.4 dB in the oral group and from 58.4 ± 15.1 dB to 22.8 ± 10.2 dB in the local group (*p* = 0.96 for post-operative comparison; *p* = 0.42 for Δ). At 1,000 Hz, thresholds improved from 60.1 ± 17.0 dB to 24.9 ± 10.3 dB in the oral group and from 58.3 ± 14.5 dB to 23.6 ± 12.1 dB in the local group (*p* = 0.62). Mean PTA-AC decreased from 60.4 ± 14.7 dB to 23.2 ± 11.8 dB in the oral group and from 56.2 ± 14.1 dB to 20.5 ± 11.6 dB in the local group, representing comparable improvements of approximately 37 dB and 36 dB, respectively (*p* = 0.70).

### 3.3 Bone-conduction thresholds

Baseline BC thresholds were comparable across most frequencies except for 250 Hz, where the local steroid group exhibited slightly higher pre-operative values (22.2 ± 14.4 dB) compared with the oral group (15.7 ± 7.8 dB, *p* = 0.017) (Table 3). Post-operatively, BC thresholds improved modestly in both groups, with no statistically significant between-group differences at any frequency.

**Table 3:** Comparison of pre-operative, post-operative, and Δ (improvement) bone-conduction thresholds between oral and local steroid groups.

Characteristic	Preoperative			Postoperative			Delta (Postop − Preop)		
	Per oral	Local	*p*	Per oral	Local	*p*	Per oral	Local	*p*
BC (250 Hz)	15.73 (7.81)	22.23 (14.36)	**0.0174**	13.05 (4.98)	14.95 (9.06)	0.2658	−2.68 (9.06)	−7.28 (15.56)	0.1215
BC (500 Hz)	20.41 (8.87)	19.64 (9.76)	0.7224	16.27 (7.19)	15.31 (7.87)	0.5800	−4.14 (9.49)	−4.33 (11.31)	0.9344
BC (1,000 Hz)	22.73 (11.11)	20.59 (9.99)	0.3798	15.97 (7.02)	17.69 (8.82)	0.3517	−6.76 (13.24)	−2.90 (12.24)	0.1908
BC (2,000 Hz)	27.84 (15.31)	24.51 (12.65)	0.3043	16.19 (9.018)	17.62 (10.39)	0.5257	−11.65 (17.09)	−6.90 (14.57)	0.1955
BC (4,000 Hz)	24.27 (14.30)	22.69 (15.26)	0.6436	16.38 (10.19)	19.36 (15.39)	0.3253	−7.89 (13.47)	−3.33 (20.84)	0.2639
BC (PTA)	24.40 (9.74)	20.58 (10.62)	0.1074	16.03 (6.61)	18.21 (7.69)	0.1888	−8.37 (11.15)	−2.37 (11.97)	**0.0268**

Data are presented as means (standard deviation) using the *t*-test for between group comparison. BC, bone conduction; PTA, pure-tone average; *p*, *p*-value.

Bold values indicate statistically significant differences between groups (*p* < 0.05).

At 1,000 Hz, post-operative BC improved to 16.0 ± 7.0 dB in the oral group and 17.7 ± 8.8 dB in the local group (*p* = 0.35). Across frequencies, the overall PTA-BC improved from 24.4 ± 9.7 dB to 16.0 ± 6.6 dB in the oral group and from 20.6 ± 10.6 dB to 18.2 ± 7.7 dB in the local group. The mean BC gain was significantly greater with oral steroid therapy (−8.4 ± 11.2 dB) compared with the local group (−2.4 ± 12.0 dB, *p* = 0.027).

### 3.4 Air–bone gap closure

Pre-operative ABG thresholds did not differ significantly between groups (Table 4). After surgery, both groups showed pronounced ABG closure across all frequencies, reflecting successful stapes replacement and effective sound transmission.

**Table 4:** Comparison of pre-operative, post-operative, and Δ (improvement) air-bone gap thresholds between oral and local steroid groups.

Characteristic	Preoperative			Postoperative			Delta (Postop − Preop)		
	Per oral	Local	*p*	Per oral	Local	*p*	Per oral	Local	*p*
ABG (250 Hz)	51.78 (13.64)	49.28 (16.11)	0.4686	24.57 (11.09)	18.97 (10.17)	0.0246	−27.21 (15.38)	−30.31 (18.19)	0.4274
ABG (500 Hz)	49.92 (14.45)	43.77 (13.75)	0.0612	21.08 (11.28)	21.59 (10.06)	0.8360	−28.84 (18.07)	−22.18 (16.51)	0.0976
ABG (1,000 Hz)	44.24 (14.21)	44.26 (17.35)	0.9971	22.51 (11.14)	21.31 (10.63)	0.6306	−21.79 (14.71)	−22.95 (18.20)	0.7498
ABG (2,000 Hz)	34.35 (16.89)	37.05 (21.06)	0.5408	19.54 (9.95)	20.18 (9.20)	0.7720	−14.81 (15.87)	−16.87 (24.24)	0.6642
ABG (4,000 Hz)	36.22 (18.35)	40.13 (17.92)	0.3502	22.30 (10.89)	18.64 (9.33)	0.1196	−13.92 (20.21)	−21.49 (21.02)	0.1142
ABG (PTA)	39.58 (14.01)	40.97 (16.25)	0.6898	20.21 (11.69)	19.13 (10.62)	0.6746	−19.37 (16.63)	−21.85 (19.41)	0.5531

Data are presented as means (standard deviation) using the *t*-test for between group comparison. ABG, air-bone gap; PTA, pure-tone average; *p*, *p*-value.

At 250 Hz, mean post-operative ABG was 24.6 ± 11.1 dB in the oral group and 19.0 ± 10.2 dB in the local group (*p* = 0.025). Although this difference favored the local group, the corresponding ΔABG values (−27.2 ± 15.4 dB vs. −30.3 ± 18.2 dB) were not significantly different (*p* = 0.43). The mean ABG-PTA improved from approximately 40 dB pre-operatively to 20 dB post-operatively in both groups, with a comparable overall reduction of nearly 20 dB (*p* = 0.55 for Δ).

## 4 Discussion

In this retrospective analysis of 76 stapedectomy patients treated with either oral prednisone or local gel-foam dexamethasone, we found broadly comparable improvements in air-conduction and air–bone gap outcomes between the two steroid protocols. The absence of major between-group differences in AC and ABG gains aligns with a growing body of literature suggesting that adjunctive steroid administration—whether systemic or local—does not substantially alter the conductive hearing outcome of stapes surgery. For example, Çelik et al. (10) reported no significant difference in postoperative AC or ABG thresholds between patients who received intraoperative IV corticosteroids and those who did not. Similarly, in a recent randomized trial of topical dexamethasone during stapedotomy, Amirzargar et al. (11) found that ABG improvement did not reach statistical significance despite a trend favoring the steroid-treated group.

The primary goal of stapedectomy is to close the air–bone gap and restore efficient sound transmission, and our results confirm that both steroid routes support this objective, with mean AC-PTA improvements of approximately 35–37 dB and ABG reductions of about 20 dB in both groups. These outcomes fall within the expected range for contemporary stapes surgery as reported in large series (12). Bone-conduction thresholds improved in both groups, with a statistically greater mean BC-PTA gain observed in the oral steroid group (−8.4 dB vs. −2.4 dB; *p* = 0.027). However, this finding should be interpreted cautiously, as the retrospective design and limited sample size preclude definitive conclusions regarding causality or clinical superiority. Moreover, most frequency-specific BC changes did not differ significantly between groups, indicating that this observation should be regarded as exploratory rather than confirmatory.

One plausible explanation for this finding is that systemic steroid exposure may provide broader inner ear protection against surgical trauma or micro-perilymphatic disturbance, whereas local application is more focused around the oval window niche (13). Animal and basic science studies suggest that dexamethasone can reduce cochlear hair cell apoptosis after trauma (14–16). If this mechanism holds in human stapes surgery, the trend we observed might reflect subtle differences in inner ear stress that are more apparent in BC thresholds than in AC or ABG.

However, the lack of statistically significant differences across most frequency-specific AC and ABG outcomes underscores that, in routine primary stapedectomy performed by an experienced surgeon, the dominant determinant of hearing improvement remains the mechanical restoration of ossicular continuity rather than the route of steroid administration. This interpretation is consistent with prior reports indicating that postoperative sensorineural deterioration is uncommon and that the overall risk–benefit profile of adjunctive steroid use in stapes surgery remains incompletely defined (17, 18).

That said, the lack of statistically significant differences across most frequency-specific AC and ABG outcomes underscores that, in routine primary stapedectomy performed by an experienced surgeon, the dominant determinant of hearing improvement remains the mechanical restoration of ossicular continuity rather than the route of steroid administration. This interpretation is consistent with prior reports indicating that postoperative sensorineural deterioration is uncommon and that the overall risk–benefit profile of adjunctive steroid use in stapes surgery remains incompletely defined. Interestingly, a prior randomized study of topical dexamethasone reported lower rates of vertigo and tinnitus with local steroid use (8, 11). The discrepancy suggests that local steroid administration may be operationally sensitive to technique, dosing, or gel-foam positioning. It also underscores the need for prospective protocols with standardized delivery and monitoring of local steroid safety.

From a mechanistic perspective, differences between systemic and local steroid administration may plausibly relate to drug distribution, duration of exposure, and penetration of cochlear structures. Systemic corticosteroids achieve more uniform inner-ear exposure via the systemic circulation and stria vascularis, potentially modulating inflammatory responses, oxidative stress, and microvascular permeability within the cochlea (6, 19). In contrast, locally applied steroids delivered via the middle ear primarily diffuse across the round and oval windows, resulting in higher perilymph concentrations near the basal turn but potentially more heterogeneous cochlear distribution (20, 21). Prior experimental and clinical studies have demonstrated that corticosteroids can attenuate cochlear inflammatory cascades and protect sensory hair cells following acoustic or surgical trauma; however, the extent to which these effects translate into clinically meaningful hearing preservation after stapes surgery remains uncertain (14, 22). In the present study, the absence of consistent frequency-specific differences and the exploratory nature of the observed bone-conduction findings suggest that any mechanistic advantage of one delivery route over the other, if present, is likely modest and warrants confirmation in prospective investigations.

The observed differences in postoperative tinnitus and infection rates may reflect factors related to local middle-ear conditions rather than a direct pharmacologic effect of steroid administration. For example, local gel-foam placement near the oval window may alter middle-ear aeration or mucosal response, potentially influencing transient auditory symptoms or local inflammatory reactions. However, given the absence of standardized documentation on symptom severity, duration, or treatment, such considerations remain speculative and should be interpreted cautiously.

In addition, although local steroid application was performed as part of routine surgical practice, procedural aspects such as gel-foam positioning and steroid distribution were not systematically documented, and their potential contribution to postoperative symptoms could not be evaluated. Prospective studies employing standardized local steroid delivery protocols may help reduce technique-related variability and clarify their impact on postoperative outcomes.

Several limitations of this study warrant acknowledgement. First, the retrospective design inherently limits causal inference and introduces a risk of selection bias, as treatment allocation was not randomized and may have been influenced by clinical, temporal, or surgeon-dependent factors not fully captured in the medical records. In addition, the presence of unmeasured confounders—such as subtle differences in disease severity, cochlear reserve, or perioperative decision-making—cannot be excluded.

Second, the relatively small sample size of 76 patients restricts statistical power, particularly for detecting modest between-group differences at individual frequencies; notably, delta bone-conduction thresholds at 2,000 Hz and 4,000 Hz differed numerically between groups but did not reach statistical significance, which may reflect insufficient power rather than true equivalence. Audiometric assessment was limited to conventional frequencies up to 4 kHz, consistent with routine postoperative evaluation following stapedectomy; extended high-frequency audiometry was not routinely performed and therefore could not be analyzed. In addition, multiple frequency-specific comparisons were performed without formal adjustment for multiple testing; these analyses should therefore be interpreted cautiously and regarded as exploratory.

Third, several baseline differences between groups may have influenced the observed outcomes. Patients in the oral steroid group were older, had a longer duration of hearing loss, and a higher prevalence of smoking, while the local steroid group included a greater proportion of patients with moderate baseline hearing loss. These imbalances may confound comparisons of postoperative hearing outcomes and complication rates. Although statistical adjustment using multivariable regression or propensity score methods could theoretically address such confounding, the relatively small sample size limited the feasibility of these approaches without risking model overfitting and unstable estimates. Consequently, these baseline differences should be considered when interpreting the findings.

Fourth, postoperative audiometric assessment was limited to a short follow-up interval of 6–12 weeks, precluding evaluation of long-term hearing stability, delayed sensorineural changes, or late complications. Fifth, although all procedures were performed by a single experienced surgeon—enhancing technical consistency—this may limit the generalizability of the findings to other surgical settings. Finally, audiometric data at frequencies above 4 kHz were not consistently available, and potential high-frequency cochlear effects could not be assessed. Collectively, these limitations indicate that the present findings should be interpreted cautiously and regarded as hypothesis-generating rather than definitive.

In this retrospective comparative study of patients undergoing primary stapedectomy for otosclerosis, both oral and local gel foam steroid administration were associated with significant postoperative improvement in air-conduction and air–bone gap thresholds, with no statistically significant difference in overall hearing gain between groups. Although greater preservation of bone-conduction thresholds was observed in the oral steroid group, this finding should be interpreted cautiously given the retrospective design and limited sample size. The higher incidence of tinnitus and infection observed in the local steroid group highlights the need for further evaluation of technique-specific factors and safety considerations. Overall, both steroid administration routes were associated with favorable short-term hearing outcomes after stapedectomy; however, larger prospective studies are required to confirm these findings and to define the optimal route and regimen for postoperative steroid therapy.

## Data Availability

The original contributions presented in the study are included in the article/Supplementary material, further inquiries can be directed to the corresponding author.
